# Neuronal Release of Cytokine IL-3 Triggered by Mechanosensitive Autostimulation of the P2X7 Receptor Is Neuroprotective

**DOI:** 10.3389/fncel.2016.00270

**Published:** 2016-11-23

**Authors:** Jason C. Lim, Wennan Lu, Jonathan M. Beckel, Claire H. Mitchell

**Affiliations:** ^1^Department of Anatomy and Cell Biology, University of PennsylvaniaPhiladelphia, PA, USA; ^2^Department of Pharmacology and Chemical Biology, University of PittsburghPittsburgh, PA, USA; ^3^Department of Physiology, University of PennsylvaniaPhiladelphia, PA, USA; ^4^Department of Ophthalmology, University of PennsylvaniaPhiladelphia, PA, USA

**Keywords:** P2X7, cytokine release, mechanosensitivity, IL-3, neuroprotection, ATP release, glaucoma, pannexin hemichannels

## Abstract

Mechanical strain due to increased pressure or swelling activates inflammatory responses in many neural systems. As cytokines and chemokine messengers lead to both pro-inflammatory and neuroprotective actions, understanding the signaling patterns triggered by mechanical stress may help improve overall outcomes. While cytokine signaling in neural systems is often associated with glial cells like astrocytes and microglia, the contribution of neurons themselves to the cytokine response is underappreciated and has bearing on any balanced response. Mechanical stretch of isolated neurons was previously shown to trigger ATP release through pannexin hemichannels and autostimulation of P2X7 receptors (P2X7Rs) on the neural membrane. Given that P2X7Rs are linked to cytokine activation in other cells, this study investigates the link between neuronal stretch and cytokine release through a P2X7-dependent pathway. Cytokine assays showed application of a 4% strain to isolated rat retinal ganglion cells (RGCs) released multiple cytokines. The P2X7R agonist BzATP also released multiple cytokines; Interleukin 3 (IL-3), TNF-α, CXCL9, VEGF, L-selectin, IL-4, GM-CSF, IL-10, IL-1Rα, MIP and CCL20 were released by both stimuli, with the release of IL-3 greatest with either stimuli. Stretch-dependent IL-3 release was confirmed with ELISA and blocked by P2X7R antagonists A438079 and Brilliant Blue G (BBG), implicating autostimulation of the P2X7R in stretch-dependent IL-3 release. Neuronal IL-3 release triggered by BzATP required extracellular calcium. The IL-3Rα receptor was expressed on RGCs but not astrocytes, and both IL-3Rα and IL-3 itself were predominantly expressed in the retinal ganglion cell layer of adult retinal sections, implying autostimulation of receptors by released IL-3. While the number of surviving ganglion cells decreased with time in culture, the addition of IL-3 protected against this loss of neurons. Expression of mRNA for *IL-3* and *IL-3Rα* increased in rat retinas stretched with moderate intraocular pressure (IOP) elevation; BBG blocked the rise in *IL-3*, implicating a role for the P2X7R in transcriptional regulation *in vivo*. In summary, mechanical stretch triggers release of cytokines from neurons that can convey neuroprotection. The enhancement of these signals *in vivo* implicates P2X7R-mediated IL-3 signaling as an endogenous pathway that could minimize damage following neuronal exposure to chronic mechanical strain.

## Introduction

Mechanical strain can trigger neuronal loss, and inflammatory signals are increasingly recognized as contributing to the response. Cytokines and chemokines have been implicated in traumatic brain injury (TBI), tumor-related swelling, increased intraocular pressure (IOP) and other pathological conditions linked to mechanical strain (Ziebell and Morganti-Kossmann, 2010; Freedman and Iserovich, 2013; Wei et al., 2014; Gyoneva and Ransohoff, 2015). Given that cytokines and chemokines can initiate both pro-inflammatory and protective responses, understanding the pathways linking mechanical strain to cytokine signaling has broad relevance for neural trauma.

Although cytokines are traditionally associated with release from cell types of the inflammatory system, they are now recognized as general signaling molecules (Iwasaki and Medzhitov, 2010; Lacy and Stow, 2011; Arango Duque and Descoteaux, 2014). Within neural tissues, microglial cells are a major source of cytokines (Hanisch, 2002), but other glial cells like astrocytes can also signal with cytokines in health and disease (Domanska et al., 2011; Kan et al., 2012; Choi et al., 2014). Neurons are typically examined as a target for cytokine signaling, with various cytokine receptors expressed on neural membranes (Bajetto et al., 2002; Gougeon et al., 2013). However, neurons are also a source of releasable cytokines; while this has been known for some time, the signaling pathways that link neural stimulation to cytokine release remain unclear (Freidin et al., 1992; Yamamoto et al., 2011).

Purinergic signaling pathways provide a likely route to connect mechanical strain with neuronal cytokine release. Throughout the body, mechanical strain leads to ATP release, with pannexin hemichannels implicated as a conduit for mechanosentitive ATP release in many cell types (Bao et al., 2004; Beckel et al., 2015; Furlow et al., 2015). Isolated neurons also respond to mechanical strain with the release of ATP through pannexin hemichannels (Xia et al., 2012). This released ATP autostimulates P2X7 receptors (P2X7Rs) on the neural membrane to elevate cytoplasmic calcium levels. Given that cytoplasmic calcium can contribute to the vesicular release of cytokines (Stow et al., 2009; Stanley and Lacy, 2010), and the stimulation of P2X7Rs is associated with cytokine release from multiple cell types (Pizzirani et al., 2007; Mingam et al., 2008; Clark et al., 2010), this study asked whether neurons could release cytokines in response to mechanical strain, whether this involved P2X7Rs, and probed the consequences of cytokine release for neuronal survival.

## Materials and Methods

### Purification of Retinal Ganglion Cells

Isolation of retinal ganglion cells (RGCs) was performed using the immunopanning procedure of Barres (Barres and Chun, 1993) as described in detail (Zhang et al., 2006). All animals were used according to protocols approved by the University of Pennsylvania Institutional Animal Care and Use Committee. In brief, retinas of Long-Evans rat pups PD 3–7 of both genders were dissected from each eye globe and digested for 30 min at 37°C in Hank’s balanced salt solution (HBSS; Gibco, Inc. Invitrogen Corp., Carlsbad, CA, USA) containing 15 U/mL papain, 0.2 mg/mL DL-cysteine and 0.004% DNase I. The retinas were washed and triturated in HBSS with 1.5 mg/mL ovomucoid, 1.5 mg/mL bovine serum albumin (BSA) and 0.004% DNase I, incubated with rabbit anti-rat macrophage antibody (10 min, 1:75, Accurate Chemical, Westbury, NY, USA) and then centrifuged at 1000 rpm for 10 min, washed and spun again. Cells were re-suspended in phosphate-buffered saline (PBS) containing 0.2 mg/mL BSA and 5 μg/mL insulin, and incubated for 15 min in a 100 mm Petri-dish coated with goat anti-rabbit IgG antibody (1:400, Jackson ImmunoResearch Inc, West Grove, PA, USA). Non-adherent cells were transferred to a second Petri-dish coated with goat anti-mouse IgM antibody (1:300, Jackson ImmunoResearch) and anti-Thy 1.1 antibody (from hybridoma T11D7e2; American Type Culture Collection, Rockville, MD, USA). After 30 min, non-adherent cells were washed off and the adherent ganglion cells were released with 0.125% trypsin for 8 min at 37°C. Enzymatic activity was neutralized using 30% fetal bovine serum in Neurobasal-A medium and the purified RGCs were collected in a centrifuge tube. The basic growth medium consisted of Neurobasal-A medium with 0.033mL/mL B-27 supplement, 3.3% rat serum, 50 ng/mL BDNF, 10 ng/mL CNTF, 50 ng/mL FGF-basic, 5 μg/mL insulin and 5 μM forskolin. Isolated RGCs were seeded onto 0.1% poly-L-lysine (Peptides International) and 1 μg/mL laminin coated coverslips or elastic silicone sheeting in stretch chambers and cultured at 37°C with 5% CO_2_.

### Cell Stretch Chamber

A specially designed stretch chamber was used to apply moderate stretch to isolated neurons as described for neuronal ATP release (Xia et al., 2012). A hole drilled in the top enclosure of the stretch chamber allowed the entry of a needle attached to a 3-way valve connected to both a 20 ml syringe, which allowed pressure elevation by injection of air, and simultaneously to a pressure transducer to monitor the pressure inside the chamber. To prevent pressure leakage, silicone grease was used to cover the hole after the needle was inserted and a layer of Teflon tape was used to screw on the top enclosure. Each stretch chamber was fitted with an elastic silicone sheet and application of 20 mmHg of pressure was calculated to result in a 4.1% deformation strain. To promote cell adhesion, the sheets were coated with 0.1% poly-L-lysine for 24 h and 1 μg/mL laminin for 2 h prior to seeding. Isolated RGCs were seeded onto silicone sheets in the stretch chamber and maintained in the basic growth medium at 37°C for 16 h. Cells were washed with low-Mg^2+^ isotonic solution containing (in mM) 105 NaCl, 5 KCl, 4 Na Hepes, 6 Hepes acid, 5 NaCO_3_, 60 mannitol, 5 glucose and 1.3 CaCl_2_ (pH 7.4). To begin the experiment, 750 μL of isotonic solution with or without P2X7R antagonists Brilliant Blue G (BBG, Sigma Chemical #B-0770 (Jiang et al., 2000)) or A438079 (Tocris, #2972 (Donnelly-Roberts and Jarvis, 2007)) were added to the stretch chambers and 30 min stabilization time was allowed prior to taking a 250 μL baseline sample. Pressure inside the stretch chamber was increased to 20 mmHg for 4 min, returned to 0 mmHg for 1 min and the cycle repeated three times for a total duration of 15 min. Immediately following stretch, a 250 μL sample of the extracellular solution was collected from the center of the stretch chamber. Samples were frozen at −20°C and used for cytokine array or Interleukin 3 (IL-3) measurement as described below.

### P2X7 Receptor Activation

Isolated RGCs were cultured for 18 h on coverslips coated with poly-L-lysine and laminin. RGCs were washed thrice with low-Mg^2+^ isotonic solution and then incubated with 1.0 mL of isotonic solution for 30 min. A 500 μL sample was collected and replaced with 500 μL of 100 μM P2X7R agonist BzATP (Young et al., 2007). After 30 min incubation in 50 μM BzATP, a 500 μL sample was collected. In some experiments 50 μM BzATP was prepared in Ca^2+^-free isotonic solution or with 10 μM P2X7R antagonist A438079 with 30 min pre-incubation in A438079. After 30 min, a sample of the extracellular solution was collected and stored at −80°C until ready for measurement.

### Cytokine Array

Samples collected before and after BzATP stimulation or samples collected from the previously detailed stretch chamber experiments were assayed for levels of cytokines using a rat antibody cytokine array (#ARY008, R&D Systems). Briefly, the experimental samples were first incubated with biotinylated detection antibodies and then incubated on nitrocellulose membranes spotted with 29 different anti-cytokine antibodies in duplicate. Cytokines present in the sample were bound to its cognate capture antibody on the membrane and the amount of attached cytokines was detected by incubating the membrane in Streptavidin-Horseradish Peroxidase (HRP) followed by chemiluminescent detection reagents (GE Healthcare). The production of light corresponding to levels of bound cytokine was determined with ImageQuant LAS4000 and the intensity of each spot was measured using ImageQuant TL analysis software (all GE Healthcare).

### Measurement of IL-3

IL-3 concentrations were determined using the Rat IL-3 ELISA kit (#CSB-E07436r, Cusabio Biotech Co., College Park, MD, USA) by following the manufacturer’s instructions. The ELISA plate provided in the kit had been coated with an IL-3 antibody, which binds IL-3 proteins present in the sample. After incubation with biotin-conjugated antibody specific for IL-3 and Avidin conjugated to HRP, the addition of a TMB (3,3′,5,5′-tetramethyl-benzidine) substrate solution produces a change in color. A sulfuric acid solution was added to stop the enzyme-substrate reaction and the plate was read using a plate reader at wavelength of 450 nm and a correction wavelength of 570 nm. IL-3 standard curves with and without drugs used in the experiment were prepared to convert absorbance values of the samples to IL-3 concentration.

### Immunoblots

Isolated cells or tissue were lysed in RIPA buffer (150 mM NaCl, 1.0% Triton X-100, 0.5% Na-Deoxycholate, 0.1% SDS, 50 mM Tris, protease inhibitor cocktail, pH 8.0). Lysates were pulse sonicated, centrifuged at 13,000 g for 10 min at 4°C and the supernatant collected and boiled with 4× sample buffer. Controls included spleen dissected from the rat pups used for ganglion cell isolation, macrophages/microglial cells as identified in the first stage of the immunopanning procedure, and rat optic nerve head (ONH) astrocytes isolated as described (Beckel et al., 2014). Protein concentration was determined by the bicinchoninic acid (BCA) assay and equal amounts of protein sample (15 μg) were separated by 10% SDS-PAGE, followed by transfer to PVDF membrane. Non-specific sites were blocked with 5% non-fat milk and incubated overnight at 4°C with mouse anti-IL-3Rα (sc-74522, 1:100, Santa Cruz Biotechnology Inc. Dallas, TX, USA). Membranes were incubated with HRP-conjugated secondary antibody (1:3000, Santa Cruz) for 1 h at room temperature (RT). Blots were developed using a chemiluminescent detection kit (Amersham) and captured using an ImageQuant LAS 4000 as described (Lu et al., 2015).

### Immunohistochemistry

Frozen retina sections (10 μm) from adult Long-Evans rats were fixed in 4% paraformaldehyde for 10 min at RT. Sections were incubated with Superblock blocking buffer (ThermoFisher Scientific# 37515) containing 10% donkey serum for 1 h at RT. The primary antibodies for IL-3 receptor alpha (1:50, mouse anti-IL-3Rα, sc-74522, Santa Cruz Biotech.) and IL-3 (1:50, goat anti-IL-3, sc-34807, Santa Cruz Biotech.) were incubated at 4°C for overnight. The secondary antibodies were conjugated to AlexaFluor 555 (1:1000, ThermoFisher Scientific) and incubated for 1 h at RT. Samples were protected from light from this point onwards. Nuclei were counterstained with 4′,6-diamidino-2-phenyindole dilactate (DAPI; Invitrogen) for 10 min at RT prior to mounting in Slow Fade Gold Antifade immunofluorescence mounting medium. Negative controls were conducted by omitting the primary antibodies. Images were visualized using a fluorescence microscope (Nikon Eclipse E600) with excitation/emission filters of 360/ >515 nm (DAPI) and 530–560/575–640 nm (AlexaFluor 555) and captured with a Retiga 2000 camera (QImaging, Surrey, BC, Canada). ImageJ^1^ (Schindelin et al., 2015) was used to subtract background, modulate intensity and combine pseudocolored images, with parallel processing for all images.

### Lactate Dehydrogenase Assay

Lactate dehydrogenase (LDH) activity released into the extracellular solution was measured as an indicator of cell membrane integrity as described previously (Guha et al., 2013), based on a coupled two-step reaction where tetrazolium salt was reduced to the colored product formazan (Cytotoxicity Detection Kit (LDH), Roche Applied Science, Indianapolis, IN, USA). Briefly, 100 μL of extracellular solution collected from cells subject to stretch or BzATP stimulation was added to a 96-well plate. A reaction mixture consisting of catalyst and dye solution was prepared and 100 μl was added to each well as per the manufacturer’s instructions. After incubation for 15–30 min at RT, the dye absorbance was measured at 490 nm using a microplate reader. Absorbance values were converted to LDH concentration by preparing LDH standards (L-LDH; from rabbit muscle, Roche) for each experiment.

### Cell Viability Assay

Cell viability was determined as previously described (Zhang et al., 2010). In brief, Long Evans rat pups (PD 3–7) were anesthetized with an intraperitoneal injection of 50/5 mg/kg ketamine/xylazine, followed by the injection of aminostilbamidine into the superior colliculus to allow for retrograde labeling of the RGCs. After allowing 2–3 days for dye transport to the ganglion cell somas in the retina, animals were sacrificed and the retinas dissected in Hank’s buffered saline solution (HBSS). The tissue was dissociated in 15 U/mL papain in HBSS at 37°C for 15 min, followed by washing in HBSS and trituration in culture medium to obtain a mixed retinal cell suspension. Cells were seeded onto coverslips coated with 0.1% poly-L-lysine and 1 μg/mL laminin. Cells were maintained at 37°C with 5% CO_2_ in culture medium with or without 10 ng/mL IL-3 (R&D Systems, Minneapolis, MN # 2524-RL) or 20 μM AG490 (Calbiochem/EMD Billerica, MA #658411). After 24 h, the cells were fixed with 4% paraformaldehyde for 15 min and the coverslips mounted onto slides with SlowFade Gold anti-fade mounting media (Invitrogen). The number of surviving ganglion cells was quantified by counting the fluorescently labeled cells in 80–120 fields observed with a 20× objective in a masked fashion.

### *In Vivo* Elevation of Intraocular Pressure

Transient non-ischemic elevation of IOP was performed based on the protocols developed by Morrison and Crowston (Morrison et al., 2014; Crowston et al., 2015). Sprague-Dawley rats (8–12 weeks) were anesthetized by intraperitoneal injection of ketamine/xylazine (100/10 mg/kg). One eye was cannulated with a 27-gauge needle (Becton Dickinson, NJ, USA) inserted into the anterior chamber and connected to a 20 ml syringe filled with sterile PBS. IOP was elevated to 50 mmHg by positioning the syringe at the appropriate height (68 cm H_2_O), while the contralateral eye without cannulation served as the normotensive control. IOP was checked with a TonoLab tonometer (Colonial Medical Supply, Franconia, NH, USA) at the beginning and end of the elevation of the reservoir. IOP was found to be remarkably consistent both throughout the 4 h of elevation and between animals. After 4 h IOP elevation, pressure was returned to normal, the needle was removed and antibiotic ointment was applied. Rats were sacrificed 24 h later and the retina dissected and processed for molecular analysis.

### Intravitreous Injection

Injections were performed under a microscope with a micropipette connected to a microsyringe (Drummond Scientific Co., Broomall, PA, USA) as described (Hu et al., 2010). P2X7R antagonist BBG (0.8%) was dissolved in sterile saline and injected 5–7 days before IOP elevation. The glass pipette filled with drug was passed through the sclera at a point approximately 1 mm from the limbus into the vitreous cavity. The total volume injected was 5 μl over a 30 s time period.

### Quantitative PCR

After enucleation, the dissected retina was immediately homogenized in 1 mL of Trizol reagent (Invitrogen Inc., Carlsbad, CA, USA). Chloroform was added, followed by centrifugation at 12,000 g for 10 min. The aqueous layer was collected and the total RNA purified using the RNeasy Mini kit (Qiagen). The High Capacity RNA-to-cDNA kit (Applied Biosystems #4387406) was used to reverse-transcribed 1 μg of total RNA to cDNA. Quantitative PCR (qPCR) was performed using a Power SYBR Green master mix (Applied Biosystems) and the quantitative assessment of gene levels was performed using a 7300 Real-Time PCR System (Applied Biosystems) as described (Reigada et al., 2005). For wells with IL-3 primers, 0.75 uL of cDNA was added per well, due to low gene expression of IL-3 in the retina. For all other wells, 0.5 uL of cDNA was added per well. Primer pair sequences used for qPCR are IL-3 F: AGTGACGACAAAGCCAATCTGAGG R: TTGTAGACACCTGGCAACACAGAGT; IL-3Rα F: AGGGAACACTGAGAGCAGGA R: TGACATCGCCTCGAACATAG; IL-3Rβ F: GGGAGGACAGCAAGACAGAG R: GGTGAGGATGAGGAAGACCA; GAPDH F: ATGACTCTACCCACGGCAAG R: TACTCAGCACCAGCATCACC. Primers were designed using Primer Blast (NCBI^2^) or Primer 3. All data were analyzed using the delta-delta Ct approach. Statistical analysis for the qPCR results was performed by comparing the change in expression of relevant genes to GAPDH, and these responses in pressurized eyes with and without antagonist BBG using a Student’s *t* test.

### Data Analysis

All data are expressed as mean ± standard error of the mean, with significance defined as *p* < 0.05. Statistical analysis for cytokine array experiments was determined using a paired *t*-test, while other data was analyzed using a one-way ANOVA followed by appropriate *post hoc* test unless otherwise noted. Analysis was performed using SigmaStat software (Systat Software, Inc., San Jose, CA, USA). % block of IL-3 expression was calculated as 100 × ([RQ_c_-RQ_b_]/RQ_c_) where RQ_c_ is expression in control and RQ_b_ is in BBG.

## Results

### Neurons Release Cytokines Following Mechanical Stretch

**Figure 1:**
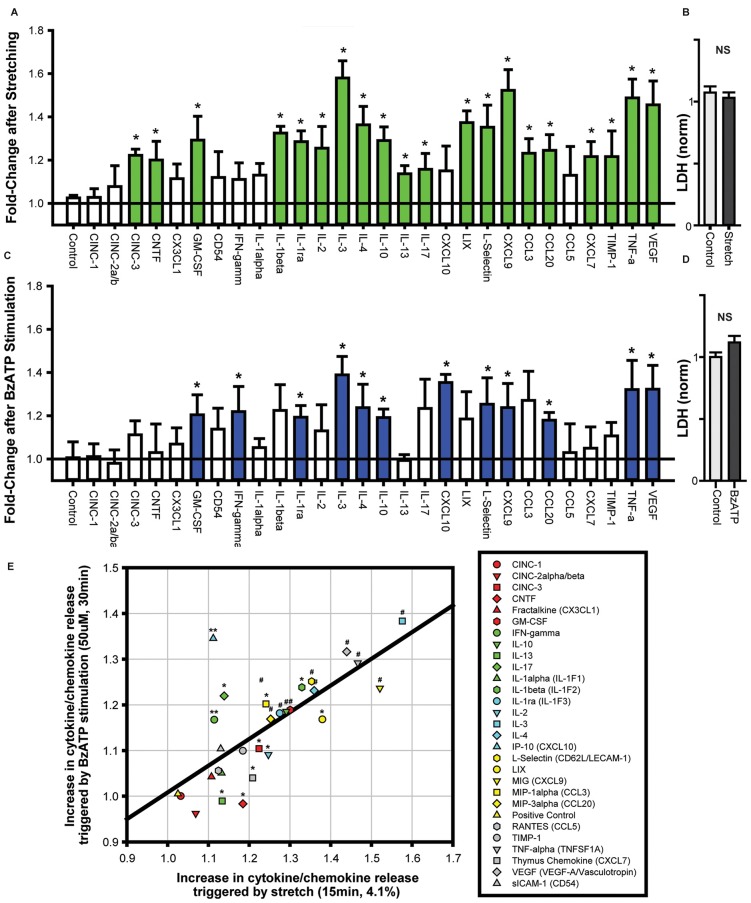
**Cytokines are released from neurons challenged with cell stretch or P2X7R stimulation. (A)** Neuronal stretching with a 4.1% strain triggered the release of numerous cytokines into the extracellular bath. Cytokines that were increased significantly are shown in green (*n* = 4, *p < 0.05, paired *t*-test). **(B)** Neuronal stretch did not increase lactate dehydrogenase (LDH) levels in the bath (NS, Not significant; *p* = 0.55, *n* = 4). **(C)** Exposure of neurons to P2X7R agonist BzATP (50 μM) for 30 min increased the level of extracellular cytokines. Cytokines that were increased significantly are shown in blue (*n* = 4, *p < 0.05, paired *t*-test). **(D)** BzATP did not increase LDH levels in the bath (*p* = 0.12, *n* = 4). **(E)** Comparison of the increase in cytokine secretion triggered by cell stretch to the increase after BzATP stimulation (For panel **E**, *p < 0.05 Stretch, **p < 0.05 BzATP, ^#^*p* < 0.05). The increase in cytokine release induced by stretch and BzATP displayed a relatively linear relationship, *r*^2^ = 0.52.

The identification of neuron-specific cytokine release required the isolation of a pure population of neurons. As the “two-step” immunopanning procedure enables the production of RGC populations of >98% purity (Zhang et al., 2006) this preparation was used to investigate neuron-specific release. The application of 20 mmHg pressure into the stretch chamber was calculated to produce a 4.1% strain on the silicone sheet (Xia et al., 2012), leading to a moderate stretch of adherent neurons. Cells were stretched for 4 min with a rest for 1 min, with three cycles applied over 15 min. This moderate stretch elevated the levels of numerous cytokines in the extracellular bath surrounding the neurons (Figure 1A). Cytokines with levels significantly higher in the bath after stretch included IL-3, TNF-α, VEGF and CXCL9. While the overall magnitude of increase was modest, this was likely due to the small number of cells secreting cytokines into a relatively large extracellular volume. Critically, extracellular LDH levels did not change following stretch (Figure 1B). This implied that the release of cytokines was a physiological response to stretch and not attributable to neuronal lysis or non-specific leakage.

As these neurons were previously shown to release ATP in response to stretch, and this released ATP was capable of autostimulating their P2X7Rs (Xia et al., 2012), the cytokine response to P2X7R stimulation was examined directly. The P2X7R agonist, BzATP (50 μM) led to a similar release of cytokines into the extracellular bath (Figure 1C). Cytokines with levels significantly higher in the bath after stretch included IL-3, TNF-α, VEGF and L-Selectin. Extracellular LDH levels were not elevated after stimulation with 50 μM BzATP (Figure 1D).

Many of the cytokines that were significantly increased by cell stretch were also elevated by BzATP stimulation. Plotting the changes in cytokine release after stretch vs. BzATP incubation illustrated a correlation between the extent of release by the two stimuli (Figure 1E). While the magnitude of release triggered by stretch was generally greater than the increase by BzATP, there was considerable similarity in the overall pattern of release for both experiments. This suggested a common mechanism of cytokine release induced by stretch and P2X7R activation from the neurons.

### Stretch-Induced Release of IL-3 Involves the P2X7 Receptor

**Figure 2:**
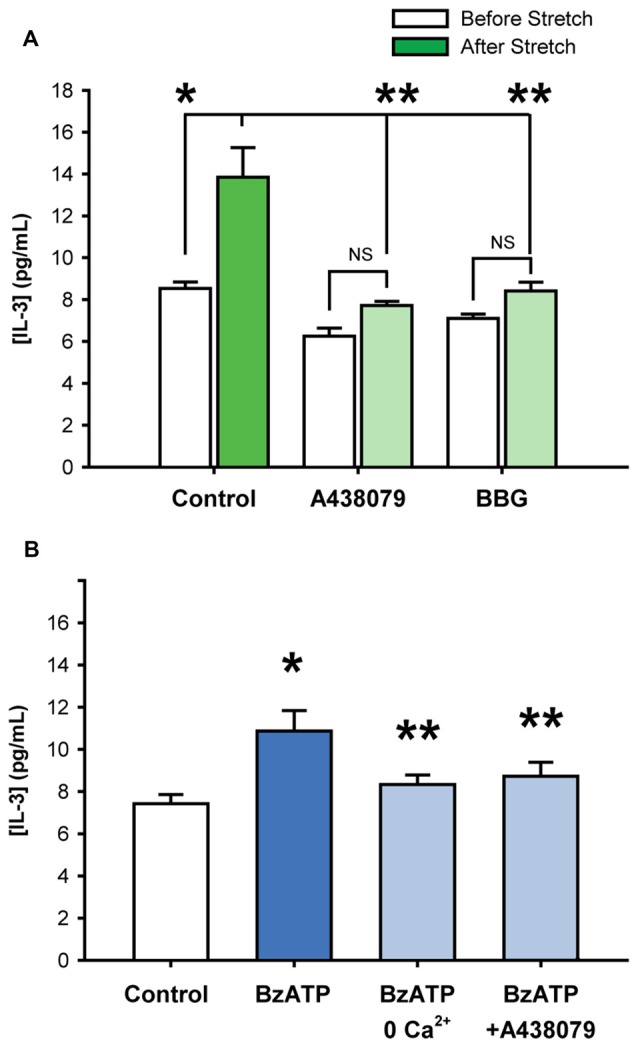
**Stretch-induced Interleukin 3 (IL-3) release involves the P2X7R. (A)** Stretching isolated neurons with 20 mmHg pressure increased levels of extracellular IL-3 (*p = 0.01, *n* = 4). The addition of P2X7R antagonists A438079 (10 μM, **p = 0.005 vs. stretched) or Brilliant Blue G (BBG, 1 μM, **p = 0.01 vs. stretched) significantly suppressed the stretch-activated release of IL-3 by 63.2 ± 7.1% and 68.2 ± 10.7%, respectively. Note that the slight inhibition of baseline IL-3 levels by A438079 and BBG were determined to be statistically NS. **(B)** Incubation of isolated neurons with 50 μM BzATP for 30 min led to a significant increase in extracellular IL-3 concentrations, compared to isotonic control. The BzATP-induced release of IL-3 was attenuated by the removal of calcium from the extracellular solution or by the presence of P2X7 receptor antagonist A438079 (10 μM). (*n* = 6, *p < 0.05 vs. Control, **p < 0.05 vs. BzATP).

While the patterns of cytokine release triggered by stretch and P2X7R activation suggested a common mechanism, additional experiments examined whether the P2X7R was involved in the stretch-mediated release. Experiments focused upon IL-3 given the relative robustness of its release from either stimuli. Baseline levels of extracellular IL-3 were 8.5 ± 0.3 pg/mL; levels rose to 13.8 ± 1.4 pg/mL after neuronal stretch, corresponding to a 1.62-fold increase in IL-3 concentration (Figure 2A). The stretch-triggered release of IL-3 was inhibited by 63.2 ± 7.1% and 68.2 ± 10.7%, respectively, by P2X7R antagonists A438079 (10 μM) or BBG (1 μM). Given that these antagonists target different sites, and that neuronal stretch released sufficient ATP to autostimulate the P2X7R on these cells (Xia et al., 2012), this strongly implicates the P2X7R in the stretch-dependent release of IL-3.

Additional evidence for the involvement of the P2X7R in the release of IL-3 was confirmed with agonist BzATP. Exposure to BzATP raised the IL-3 concentration surrounding the neurons from 7.4 ± 0.4 pg/mL to 10.9 ± 1.0 pg/mL (Figure 2B). The absence of calcium in the bath solution significantly reduced the release of IL-3 by BzATP to 8.3 ± 0.4 pg/mL. The P2X7R antagonist A438079 (10 μM) also attenuated the BzATP-triggered IL-3. This strongly suggested that BzATP was acting at P2X7Rs and not other receptor types.

### IL-3 Stimulation is Neuroprotective

**Figure 3:**
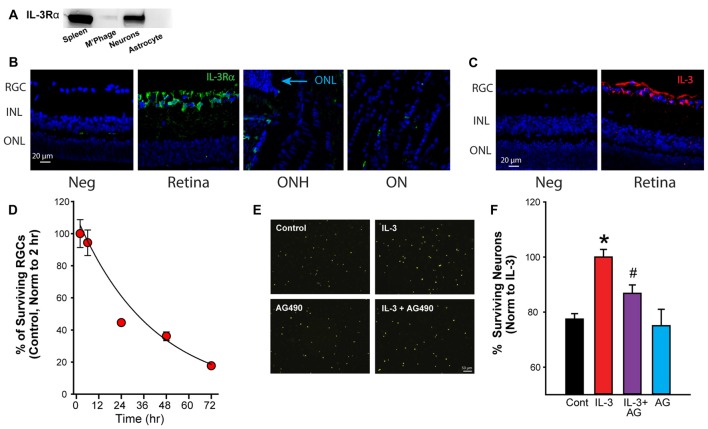
**Activation of the IL-3 receptor is neuroprotective. (A)** Immunoblot showing presence of IL-3 receptor α (IL-3Rα) in immunopanned ganglion cells, but not astrocytes. Bands for IL-3Rα were also detected on material from spleen but not macrophages/microglia (M’Phage). **(B)** Staining for IL-3Rα (green) was found primarily in the RGC layer in adult retinas. Blue—4′,6-diamidino-2-phenyindole dilactate (DAPI). Neg- no primary antibody, RGC, retinal ganglion cell layer; INL, inner nuclear layer; ONL, outer nuclear layer; ONH, optic nerve head; ON, optic nerve. **(C)** Staining for cytokine IL-3 (red) was also found predominantly in the RGC layer of the retina. **(D)** Percentage of ganglion cells decreases with time in culture. Numbers normalized to levels 2 h after plating (*n* = 3). **(E)** Images of fluorescently labeled neurons (ex 360 nm). Neurons in mixed culture had been incubated with growth medium (Control), IL-3 (10 ng/ml), IL-3 + AG490 (20 μM) or AG490 alone for 24 h to determine the effects of IL-3 on neuronal survival. **(F)** Quantification of effect of IL-3 on cell survival. IL-3 increased the number of surviving RGCs (labeled with fluorescent marker fluorogold) after 24 h in culture (*p = 0.003 IL-3 vs. control; ^#^*p* < 0.05); the number of surviving cells decreases over this period in untreated conditions. The addition of JAK2 inhibitor AG490 reduced the neuroprotective effects of IL-3 (*p* = 0.027 IL-3 vs. AG490 + IL-3, *n* = 5–11, normalized to levels in IL-3).

While the results above suggest the mechanosensitive release of IL-3 depended upon a P2X7R-dependent pathway, additional experiments probed the possible targets and consequences of IL-3. Western blot analysis revealed that the ligand-binding alpha subunit of the IL-3 receptor (IL-3Rα) was expressed by RGCs, but not by ONH astrocytes (Figure 3A). Immunohistochemistry identified RGCs as the primary of location for both IL-3Rα (Figure 3B) and IL-3 (Figure 3C) staining in the posterior eye of adult rats. Together, this implied the IL-3 released by ganglion cells autostimulated their own receptors.

Given reports of IL-3 as neuroprotective (Zambrano et al., 2007; Luo et al., 2012), the effect of IL-3 on neuronal survival was investigated. When grown in standard medium, the number of RGCs reduced with time (Figure 3D). However, incubation with IL-3 increased the number of surviving cells over 24 h (Figure 3E). As IL-3 activation can involve the JAK/STAT signaling pathway (Reddy et al., 2000), the effect of the JAK2 inhibitor AG490 was examined. The number of surviving neurons in the presence of IL-3 + 20 μM AG490 was reduced. When numbers were quantified across multiple trials, IL-3 increased the number of surviving neurons after 24 h in culture by 24% (Figure 3F), while AG490 reduced the protection by 54%. Incubating the cells with AG490 alone did not affect the neuronal survival.

### Pressure-Dependent Rise in IL-3 *In Vivo* Needs P2X7R

**Figure 4:**
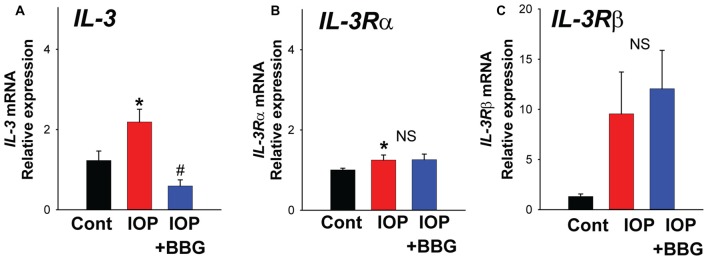
**Change in IL-3 expression *in vivo*. (A)** Quantitative PCR (qPCR) for *IL-3* indicating increased expression in rat retina following non-ischemic elevation in intraocular pressure (IOP) to 50 mmHg for 4 h (IOP). Intravitreal injection of P2X7R antagonist BBG (0.8%) 5–7 days before the elevation of IOP (IOP + BBG) prevented this rise. *p = 0.010 vs. normal IOP. ^#^*p* = 0.002 IOP vs. IOP + BBG. **(B)** Expression of *IL-3Rα* receptor was increased following increased IOP (*p* = 0.019) but BBG did not change expression. **(C)** Levels of receptor *IL-3Rβ* mRNA rose but this was NS. *N* = 5 each for pressure vs. control and BBG + pressure vs. control.

To determine whether IL-3 signaling was involved in the response to mechanical strain *in vivo*, the effect of a non-ischemic elevation of IOP was examined. Pressure was raised to 50 mm Hg for 4 h. There was a significant rise in mRNA for *IL-3* (Figure 4A) and the receptor *IL-3Rα* in retinas exposed to elevated IOP (Figure 4B). The mRNA for the *IL-3Rβ* isoform increased 9.6 ± 4.6 fold but this was not significant (NS; Figure 4C). Intravitreal injection of P2X7R antagonist BBG significantly reduced the rise in IL-3 expression, blocking the levels by 72.8 ± 7.8% (*N* = 5). BBG had no discernable effect on levels of IL-3Rα or IL-3Rβ.

## Discussion

**Figure 5:**
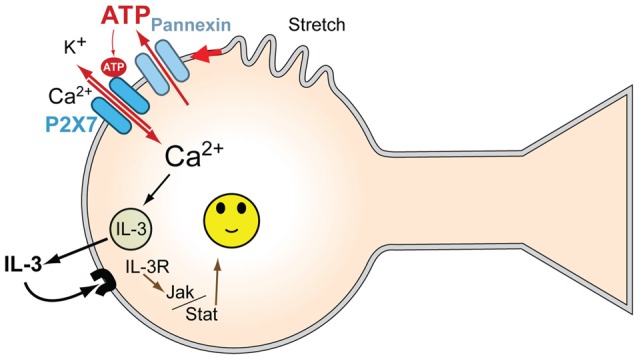
**Model for mechanosensitive P2X7/IL-3 signaling in neurons.** Mechanical strain of the neurons leads to ATP release through pannexin hemichannels; this released ATP autostimulates P2X7Rs, leading to an influx of Ca^2+^. P2X7R activation leads to the release of multiple cytokines, including IL-3, dependent upon this Ca^2+^ influx. The released IL-3 autostimulates IL-3Rα/IL-3Rβ receptors on the neurons, providing neuroprotection via JAK2 signaling pathways. Together, this provides a signaling pathway to protect neurons from moderate mechanical strain and stretch.

This study demonstrates that moderate stretch triggers the release of multiple cytokines from isolated neurons. A parallel cytokine release is produced by exposing the cells to P2X7R agonist BzATP. The correlation between the release profiles triggered by stretch and P2X7R stimulation suggested that the cytokine release pathways were linked. IL-3 showed the largest proportional release by either stimuli, and the ability of two different P2X7R antagonists to inhibit the stretch-dependent IL-3 release strongly suggested the release required P2X7R stimulation. We have previously demonstrated that stretch initiates a release of ATP through pannexin hemichannels from these neurons capable of autostimulating their P2X7R; the ability of apyrase to prevent swelling activated currents through the P2X7R, to reduce the regulatory volume decrease accompanying swelling, and to block the rise in intracellular calcium that followed swelling confirmed the central role of released extracellular ATP in the stimulation of this P2X7 receptor (Xia et al., 2012). The stretch-dependent release of IL-3 is consistent with this release of ATP and autostimulation of the P2X7R (Figure 5).

This study identified several cytokines significantly released by both moderate stretch and BzATP including IL-3, TNF-alpha, CXCL9, VEGF, L-selectin, IL-4, GM-CSF, IL-10, IL1Ra, MIP and CCL20. Mechanistic evaluation was focused on IL-3 because it showed the most robust release with both stimuli. IL-3 is expressed in neuronal populations (Konishi et al., 1994) and its activity is mediated through cell surface receptors composed of a ligand-binding IL-3Rα subunit and transmembrane IL-3Rβ subunit (Appel et al., 1995). IL-3 exhibits predominantly neuroprotective actions; it protects cortical neurons from amyloid peptide-induced death (Zambrano et al., 2007) and protects neuroprogenitor and adult neurons cells (Luo et al., 2012). It promotes the survival of sensory neurons and triggers the formation of a neural net (Moroni and Rossi, 1995) and rescues hippocampal neurons from ischemic injury (Wen et al., 1998). Results from the present study suggest IL-3 can also protect RGCs from death. As the Jak/Stat pathway was previously shown to be activated following stimulation of the IL-3 receptor (Reddy et al., 2000) and is associated with increased protection of neurons in general, and of RGCs in particular (Peterson et al., 2000; Wang et al., 2015), involvement of this pathway is a reasonable hypothesis, although this awaits further confirmation.

The cytokine release measurements in this study were performed on isolated neurons from neonatal rats as they survive manipulation more robustly than adult cells. However, experiments from cells in adult rats exposed to mechanical stretch following a moderate elevation of IOP suggest IL-3 signaling may be relevant in adults too. The expression of IL-3 and IL-3Rα was largely restricted to the ganglion cell layer in adult retina. The rise in mRNA expression for both the cytokine *IL-3* and the receptor subunit *IL-3Rα* upon exposure to elevated pressure suggests the recruitment of the signaling system upon mechanical strain. The ability of P2X7R antagonist BBG to prevent upregulation of *IL-3* suggests P2X7R involvement in both the transcription and release of IL-3. Our preliminary results have identified a similar role for the P2X7 receptor in the upregulation IL-6 dependent on NKκB (Lu et al., 2013). Stimulation of the P2X7R leads to the nuclear translocation of NKκB in osteoclasts (Korcok et al., 2004), while co-immunopreciptation indicates the C-terminal of the P2X7 receptor physically contacts MyD88 upstream of NKκB (Liu et al., 2011). The mechanisms linking P2X7R activation to priming of IL-3 in neurons remain to be determined, but these reports confirm the ability of the P2X7R to activate key components in the pathway.

While the absolute levels of IL-3 released from the isolated ganglion cells into the bath are low, previous experience with ATP release suggests the levels sampled in the bath represent a 1000-fold dilution over that close to the cells. The mechanosensitive release of ATP from these neurons makes it critical to ensure the sampling probe does not touch the cells, necessitating the use of a considerable extracellular volume (Xia et al., 2012). Although the absolute concentration of ATP measured in the bath after cell stretch using this system was 10 nM, we demonstrated that the released ATP was capable of autostimulating the P2X7 receptor with measurements from patch clamp recordings, cytoplasmic calcium imaging and regulatory volume decrease analysis (Xia et al., 2012). Of note, the EC_50_ of ATP at the rat P2X7R is approximately 100 μM (Bianchi et al., 1999), i.e., four orders of magnitude greater than the levels of ATP measured in the bath. Direct measurement of released ATP levels on the cell surface with attached luciferase show a similar dilution (Beigi et al., 1999). It is thus not unreasonable to expect IL-3 detected in the bath at 10 pg/ml to correspond to 10 ng/ml close to the cell membrane. As this is the level of IL-3 capable of protecting the neurons, the concentrations required for autostimulation are reasonable. It is worth pointing out that the lack of detectable LDH was not affected by this bath dilution, as our studies have demonstrated cell lysis leads to a substantial and readily detectable LDH signal in the bath, while stretch or swelling does not (Beckel et al., 2014).

### Physiological Implications

While the loss of RGCs is associated with sustained IOP elevation in glaucoma, the neurons can survive for a considerable time before their eventual death. In addition, certain conditions, such as advancing age, can make ganglion cells more susceptible to death when exposed to the same pressure rise in some models (Kim et al., 2004; Steinhart et al., 2014). Understanding the endogenous mechanisms that minimize the pathological effects of increased mechanical strain on the neurons is a key target in preventing their loss in disease. Although aberrant immune signaling has been implicated in the pathogenesis of glaucoma (Tezel and Wax, 2004; Grus et al., 2008; Wax and Tezel, 2009; Križaj et al., 2014), certain cytokines have also been recognized for their ability to protect ganglion cells (Sholl-Franco et al., 2001; Sappington et al., 2006). It is tempting to propose that the increased expression of mRNA for *IL-3* and *IL-3Rα* in eyes exposed to moderate IOP elevation may be part of an endogenous protective response, although this requires further confirmation.

### Linking Mechanical Stain with Cytokine Release Through Pannexin/P2X7R Interactions

The potential neuroprotective actions of the P2X7R are at odds with its traditional identification as a “death receptor”, but reflect a growing acknowledgment that expression of the receptor in stable adult neurons can actually be beneficial. Pannexin hemichannels frequently act as a conduit for the mechanosensitive release of ATP (Bao et al., 2004) and thus provide a mechanism to activate P2 signaling after swelling or stretch (Wicki-Stordeur and Swayne, 2014). Mechanical stimulation of the neurons in the present study activated ATP release through pannexins that autostimulates adjacent P2X7Rs. While pannexin-mediated stimulation of P2X7Rs can activate inflammatory pathways such as the NLRP3 inflammasome and lead to neuronal death (Gulbransen et al., 2012), the autostimulation of P2 receptors following pannexin activation can prevent apoptosis linked to mechanical deformation (Furlow et al., 2015). Clearly the consequences of pannexin/P2 signaling are context dependent and may differ in the varied purinergic signaling pathways throughout the retina and brain (Burnstock, 2013; Sanderson et al., 2014).

Finally, it should be emphasized that while the current study pertains most directly to the loss of RGCs following the rise in pressure accompanying glaucoma, the findings also have general relevance to the inflammatory responses in neural tissues after TBI. Of particular relevance may be the upregulation of genes linked to survival in astrocytes after increased cranial pressure included IL-3 (Vandevord et al., 2008). Whether this is part of a pannexin/P2X7 signaling complex, as it seems to be in the retina, remains to be determined.

## Author Contributions

JCL helped design the study, carried out most of the *in vitro* experiments, performed analysis of molecular and protein data and immunohistochemistry. WL performed experiments associated with the elevation of IOP and immunohistochemical staining on rats and analyzing associated data. JMB contributed to the experimental design and interpretation of data. CHM conceived of the study, and participated in its design and coordination and helped to draft the manuscript. All authors were involved in critical revisions of the work. All authors also approve of the final version to be published and agree to be accountable for all aspects of the work in ensuring that questions related to the accuracy or integrity of any part of the work are appropriately investigated and resolved.

## Funding

This work is supported by grants from the NIH EY015537 and EY013434 and core grant EY001583 (CHM), NIH DK106115 (JMB) and the Jody Sack Fund (WL). These funding bodies had no direct role in the design of the study and collection, analysis and interpretation of data or and in writing the manuscript.

## Conflict of Interest Statement

The authors declare that the research was conducted in the absence of any commercial or financial relationships that could be construed as a potential conflict of interest.

## Footnotes

^1^https://imagej.nih.gov/ij/

^2^http://www.ncbi.nlm.nih.gov/tools/primer-blast/
